# Correction: Dominguez-Meijide et al. Pharmacological Modulators of Tau Aggregation and Spreading. *Brain Sci.* 2020, *10*, 858

**DOI:** 10.3390/brainsci14090909

**Published:** 2024-09-09

**Authors:** Antonio Dominguez-Meijide, Eftychia Vasili, Tiago Fleming Outeiro

**Affiliations:** 1 Department of Experimental Neurodegeneration, Center for Biostructural Imaging of Neurodegeneration, University Medical Center Goettingen, 37073 Goettingen, Germany; antonio.meijide@usc.es (A.D.-M.); evassili@gmail.com (E.V.); 2 Laboratory of Neuroanatomy and Experimental Neurology, Dept. of Morphological Sciences, CIMUS, IDIS, University of Santiago de Compostela, 15782 Santiago de Compostela, Spain; 3 Max Planck Institute for Experimental Medicine, 37075 Goettingen, Germany; 4 Translational and Clinical Research Institute, Faculty of Medical Sciences, Newcastle University, Framlington Place, Newcastle Upon Tyne NE2 4HH, UK

There was an error in the original publication [1]. References 34, 41, 52, 53, 56, 61, 64, 65, 68, 69, 71, 72, 74, 75, 76, 77, 81, 85, 86, 110, 112, and 114 were not the ones intended, and were unrelated with the main text. A correction has been made to References 34, 41, 52, 53, 56, 61, 64, 65, 68, 69, 71, 72, 74, 75, 76, 77, 81, 85, 86, 110, 112, and 114, and appear as follows:

34. Chaudhary, A.R.; Berger, F.; Berger, C.L.; Hendricks, A.G. Tau directs intracellular trafficking by regulating the forces exerted by kinesin and dynein teams. *Traffic* **2018**, *19*, 111–121.
41. Lim, S.; Haque, M.M.; Kim, D.; Kim, D.J.; Kim, Y.K. Cell-based Models To Investigate Tau Aggregation. *Comput. Struct. Biotechnol. J.* **2014**, *12*, 7–13.
52. Lee, V.M.; Goedert, M.; Trojanowski, J.Q. Neurodegenerative tauopathies. *Annu. Rev. Neurosci.* **2001**, *24*, 1121–1159.
53. Rademakers, R.; Cruts, M.; van Broeckhoven, C. The role of tau (MAPT) in frontotemporal dementia and related tauopathies. *Hum. Mutat.* **2004**, *24*, 277–295.
56. Cowan, C.M.; Mudher, A. Are tau aggregates toxic or protective in tauopathies? *Front. Neurol.* **2013**, *4*, 114.
61. Furcila, D.; Domínguez-Álvaro, M.; DeFelipe, J.; Alonso-Nanclares, L. Subregional Density of Neurons, Neurofibrillary Tangles and Amyloid Plaques in the Hippocampus of Patients With Alzheimer’s Disease. *Front. Neuroanat.* **2019**, *13*, 99.
64. He, Z.; Guo, J.L.; McBride, J.D.; Narasimhan, S.; Kim, H.; Changolkar, L.; Zhang, B.; Gathagan, R.J.; Yue, C.; Dengler, C.; et al. Amyloid-β plaques enhance Alzheimer’s brain tau-seeded pathologies by facilitating neuritic plaque tau aggregation. *Nat. Med.* **2018**, *24*, 29–38.
65. Park, S.; Lee, J.H.; Jeon, J.H.; Lee, M.J. Degradation or aggregation: The ramifications of post-translational modifications on tau. *BMB Rep.* **2018**, *51*, 265–273, Erratum in *BMB Rep.* **2020**, *53*, 391.
68. Elbaum-Garfinkle, S.; Rhoades, E. Identification of an aggregation-prone structure of tau. *J. Am. Chem. Soc.* **2012**, *134*, 16607–16613.
69. Lee, V.M.; Balin, B.J.; Otvos, L., Jr.; Trojanowski, J.Q. A68: A major subunit of paired helical filaments and derivatized forms of normal tau. *Science* **1991**, *251*, 675–678.
71. Weissmann, C.; Reyher, H.J.; Gauthier, A.; Steinhoff, H.J.; Junge, W.; Brandt, R. Microtubule binding and trapping at the tip of neurites regulate tau motion in living neurons. *Traffic* **2009**, *10*, 1655–1668.
72. Lathuilière, A.; Valdés, P.; Papin, S.; Cacquevel, M.; Maclachlan, C.; Knott, G.W.; Muhs, A.; Paganetti, P.; Schneider, B.L. Motifs in the tau protein that control binding to microtubules and aggregation determine pathological effects. *Sci. Rep.* **2017**, *7*, 13556.
74. Barthélemy, N.R.; Li, Y.; Joseph-Mathurin, N.; Gordon, B.A.; Hassenstab, J.; Benzinger, T.L.; Buckles, V.; Fagan, A.M.; Perrin, R.J.; Goate, A.M.; et al. A soluble phosphorylated tau signature links tau, amyloid and the evolution of stages of dominantly inherited Alzheimer’s disease. *Nat. Med.* **2020**, *26*, 398–407.
75. Mocanu, M.M.; Nissen, A.; Eckermann, K.; Khlistunova, I.; Biernat, J.; Drexler, D.; Petrova, O.; Schönig, K.; Bujard, H.; Mandelkow, E.; et al. The potential for beta-structure in the repeat domain of tau protein determines aggregation, synaptic decay, neuronal loss, and coassembly with endogenous Tau in inducible mouse models of tauopathy. *J. Neurosci.* **2008**, *28*, 737–748.
76. Olsen, M.; Aguilar, X.; Sehlin, D.; Fang, X.T.; Antoni, G.; Erlandsson, A.; Syvänen, S. Astroglial Responses to Amyloid-Beta Progression in a Mouse Model of Alzheimer’s Disease. *Mol. Imaging Biol.* **2018**, *20*, 605–614.
77. Valotassiou, V.; Malamitsi, J.; Papatriantafyllou, J.; Dardiotis, E.; Tsougos, I.; Psimadas, D.; Alexiou, S.; Hadjigeorgiou, G.; Georgoulias, P. SPECT and PET imaging in Alzheimer’s disease. *Ann. Nucl. Med.* **2018**, *32*, 583–593.
81. Ferrer, I.; Andrés-Benito, P.; Zelaya, M.V.; Aguirre, M.E.E.; Carmona, M.; Ausín, K.; Lachén-Montes, M.; Fernández-Irigoyen, J.; Santamaría, E.; Del Rio, J.A. Familial globular glial tauopathy linked to MAPT mutations: Molecular neuropathology and seeding capacity of a prototypical mixed neuronal and glial tauopathy. *Acta. Neuropathol.* **2020**, *139*, 735–771.
85. Novak, P.; Cehlar, O.; Skrabana, R.; Novak, M. Tau Conformation as a Target for Disease-Modifying Therapy: The Role of Truncation. *J. Alzheimers Dis.* **2018**, *64*, S535–S546.
86. Amadoro, G.; Latina, V.; Corsetti, V.; Calissano, P. N-terminal tau truncation in the pathogenesis of Alzheimer’s disease (AD): Developing a novel diagnostic and therapeutic approach. *Biochim. Biophys. Acta. Mol. Basis Dis.* **2020**, *1866*, 165584.
110. Maeda, S.; Sahara, N.; Saito, Y.; Murayama, M.; Yoshiike, Y.; Kim, H.; Miyasaka, T.; Murayama, S.; Ikai, A.; Takashima, A. Granular tau oligomers as intermediates of tau filaments. *Biochemistry* **2007**, *46*, 3856–3861.
112. Michel, C.H.; Kumar, S.; Pinotsi, D.; Tunnacliffe, A.; St George-Hyslop, P.; Mandelkow, E.; Mandelkow, E.M.; Kaminski, C.F.; Kaminski Schierle, G.S. Extracellular monomeric tau protein is sufficient to initiate the spread of tau protein pathology. *J. Biol. Chem.* **2014**, *289*, 956–967.
114. Weaver, C.L.; Espinoza, M.; Kress, Y.; Davies, P. Conformational change as one of the earliest alterations of tau in Alzheimer’s disease. *Neurobiol. Aging* **2000**, *21*, 719–727.

The authors state that the scientific conclusions are unaffected. This correction was approved by the Academic Editor. The original publication has also been updated.

## References

[1] Dominguez-Meijide A., Vasili E., Outeiro T.F. (2020). Pharmacological Modulators of Tau Aggregation and Spreading. Brain Sci..

